# ItemSight: Development of a Shiny Application for Simplified Item Analysis of Type A Multiple-Choice Questions

**DOI:** 10.7759/cureus.109190

**Published:** 2026-05-19

**Authors:** Himel Mondal, Pradosh Kumar Sarangi, Shaikat Mondal, Anupkumar D Dhanvijay, Rintu K Gayen, Aritra Mukhopadhyay, Raunak Mishra

**Affiliations:** 1 Physiology, All India Institute of Medical Sciences, Deoghar, Deoghar, IND; 2 Radiodiagnosis, All India Institute of Medical Sciences, Deoghar, Deoghar, IND; 3 Physiology, Raiganj Government Medical College and Hospital, Raiganj, IND; 4 Electronics and Communication Engineering, Institute of Engineering and Management, Kolkata, IND

**Keywords:** educational assessment, item analysis, multiple-choice questions, reliability, shiny application, test quality

## Abstract

Item analysis is a vital step in educational assessment to check the quality of multiple-choice questions (items). Traditional item analysis methods using spreadsheets or statistical software are often time-consuming, error-prone, and challenging for educators with limited programming expertise. To address these issues, ItemSight, an intuitive software for item analysis, was developed to automate and simplify the psychometric evaluation of test items. The software was developed using the R-Shiny framework. It computes item difficulty, discrimination index, distractor efficiency, internal consistency (if an item is deleted), and point-biserial correlation. To run the software, users need R and RStudio with installed packages and the code we provide. After analysis, the report can be easily exported in comma-separated values (CSV) format for further analysis. All data remains stored locally on the user's computer, ensuring privacy. Currently, the software supports only type A multiple-choice questions (four options with a single best answer). It can be accessed and used through RStudio without any cost. The tool serves as a valuable resource for educators and researchers in medical and allied health sciences, promoting evidence-based improvement of assessment quality.

## Introduction

Item analysis is a systematic approach used to evaluate the quality and performance of examination questions, particularly multiple-choice questions (MCQs), which are widely used in medical education because of their objectivity, reliability, and ability to assess a broad range of knowledge domains [1,2]. In item analysis, several psychometric indicators are examined to determine whether a question functions appropriately. The difficulty index reflects the proportion of students answering an item correctly, indicating whether the question is too easy or too difficult. The discrimination index indicates how effectively an item differentiates between high- and low-performing students, while reliability coefficients assess the internal consistency of the overall examination [3,4]. For example, a question answered correctly by nearly all students may contribute little to distinguishing competency levels, whereas a question predominantly answered correctly by high-performing students is generally considered a high-quality item.

High-quality MCQs are essential because assessment outcomes directly influence student progression, competency evaluation, curriculum review, and accreditation standards in medical education [5]. Poorly constructed questions may produce misleading estimates of student ability, compromise examination validity, and result in unfair pass-fail decisions. Item analysis helps educators identify flawed or ambiguous questions, revise ineffective distractors, and improve the overall quality of assessment programs [6,7].

Despite its recognized importance, the routine implementation of item analysis remains challenging in many educational settings. Previous studies have identified barriers such as limited faculty training in psychometrics, insufficient time, lack of institutional support, and difficulties in using statistical software for routine assessment evaluation [8]. In practice, educators often conduct item analysis using spreadsheet software such as Microsoft Excel or dedicated psychometric software. However, spreadsheet-based workflows typically require manual formula entry and repetitive data handling, increasing the risk of calculation errors and making the process time-consuming. Advanced statistical software may offer comprehensive analytical capabilities, but it often requires licensing costs and familiarity with statistical procedures, limiting accessibility for educators with limited technical expertise [9].

To address these challenges, we developed ItemSight, an interactive R Shiny-based application designed to automate item analysis within a user-friendly interface. The application enables educators to calculate and interpret commonly used psychometric indices without requiring advanced statistical knowledge, thereby facilitating routine quality assessment of MCQs in medical education.

This article was presented at the 12th State Association of Physiologists and Pharmacologists of India Conference (APPICON) 2026, conducted on February 6-7, 2026, at Calcutta National Medical College and Hospital, Kolkata, India.

## Technical report

Parts of MCQ

A well-constructed MCQ consists of several key parts that together ensure clarity and effectiveness in assessment. The parts are described briefly in Table 1 [10].

**Table 1:** Parts of a multiple-choice question This is an example of a multiple-choice question with a single best answer and three distractors.

Part	Description	Example
Stem	The question or statement that presents the problem or task to be answered	A 25-year-old male presents with a sudden onset of chest pain and shortness of breath after lifting heavy weights. On examination, he has decreased breath sounds on the right side. A chest X-ray reveals a collapsed right lung with a visible pleural line.
Lead-in	The specific question or instruction directing the student what to do (sometimes part of the stem)	What is the most likely diagnosis?
Options/choices	The set of possible answers provided, including one correct answer and several distractors	A. Pulmonary embolism B. Acute myocardial infarction C. Spontaneous pneumothorax D. Bronchial asthma
Key/correct answer	The single best or correct option among the choices	C. Spontaneous pneumothorax
Distractors	The incorrect but plausible options are designed to distract those who do not know the correct answer	A. Pulmonary embolism B. Acute myocardial infarction D. Bronchial asthma

Item analysis metrics

The item analysis metrics that form the basis of the Shiny application's calculation methods are shown in Table 2 and described below.

**Table 2:** Calculation method of item analysis metrics This is only for multiple-choice questions with four response options (three distractors and one answer key).

Metric	Formula/method	Explanation
Difficulty index (p-value)	Number of correct responses ÷ total number of students × 100	Percentage of students who answered the item correctly
Difficulty index (27% method)	(H + L) ÷ N × 100	H = correct answers in top 27% scorers; L = correct answers in bottom 27% scorers; N = total students in upper and lower 27%
Discrimination index	2 × (H – L) ÷ N	
Distractor efficiency	Functional distractors ÷ 3 × 100	A distractor is "functional" if selected by at least 5% of students
Point-biserial correlation	Correlation between item score (0 or 1) and total test score	Pearson's correlation with one dichotomous variable
Cronbach's alpha	(k ÷ (k – 1)) × (1 – (sum of item variances ÷ variance of total scores))	k = total number of items

Difficulty Index (Proportion Correct/P-Value)

The difficulty index is calculated as the proportion of students who answered an item correctly. It is obtained by dividing the number of correct responses by the total number of students and expressing it as a percentage [11]. This measure is calculated to know how easy or difficult an item is. A higher value means the item is easier, while a lower value indicates more difficulty. It gives us an idea of whether the item is appropriate for the level of students being assessed.

Difficulty Index (27% Method)

This method compares item performance between the top 27% of scorers and the bottom 27% [12]. The number of correct responses in both groups is added and divided by the total number of students in these groups. This calculation is used because extreme groups provide a clearer picture of how well an item differentiates ability levels. It gives us an idea of the item's effectiveness at distinguishing stronger from weaker students. This method is more commonly used.

Discrimination Index

The discrimination index is calculated as the difference in correct responses between the top 27% and bottom 27% groups, standardized by the total number of students in those groups [13]. It is calculated to see how well an item separates high-performing from low-performing students. A high positive value means the item is good at discrimination, while a negative value suggests a faulty question. This gives us an idea about the quality of the item in terms of its ability to differentiate student performance.

Distractor Efficiency

Distractor efficiency is based on how many of the wrong options (distractors) are chosen by at least 5% of students [14]. The percentage of functional distractors is calculated for each item. It is calculated to check if the incorrect options are plausible enough to attract students who do not know the answer. This gives us an idea of whether distractors are effective in testing knowledge or if they are too obvious to be avoided.

Point-Biserial Correlation

The point-biserial correlation is the Pearson correlation between the item score (0 for wrong; 1 for correct) and the total test score [15]. It is calculated to measure the consistency of an item with the overall performance of students. A high positive correlation means that students who did well overall also got this item correct, indicating a good item. This gives us an idea about the alignment of the item with the overall test quality.

Cronbach's Alpha (and Alpha if Item Deleted)

Cronbach's alpha is calculated using item variances and the variance of the total test score. It measures the internal consistency or reliability of the test as a whole. It is calculated to assess whether the items collectively measure the same construct. A higher alpha indicates more reliable items [16]. The "alpha if item deleted" shows how the overall reliability would change if one item were removed, giving us an idea of which items weaken the test's consistency. In item analysis, the Kuder-Richardson Formula 20 (KR-20) is used for measuring the reliability [2]. If the data is dichotomous (1 and 0 in the MCQ score), then both yield the same result [17]. In this application, Cronbach's alpha is being calculated.

Development of the application

The ItemSight application was developed using the R programming language and the Shiny framework to provide an interactive and accessible platform, and it is being utilized in biomedical application development [18]. The app integrates several R packages, including Shiny for building the user interface and server logic, DT for rendering interactive tables, dplyr, and tidyr for efficient data manipulation. The code of the application is available from Mendeley Data [19].

Ten sets of MCQ response sheets were created to evaluate the software's performance. Each set contained varying numbers of student responses (100-250) and MCQ items (20-100). For every dataset, both the students' MCQ answers and the corresponding answer keys were entered into the software. To assess accuracy, five of the 10 response sets were also scored manually using the same answer keys. Manual calculations were independently performed and cross-verified by the authors, who have prior experience (>5 years) in medical education research, psychometric assessment, and item analysis methodology. Since the application is based on established mathematical formulas with deterministic outputs, the possibility of computational error is virtually negligible when calculations are performed correctly. The manually derived item-wise scores were then compared with the software-generated outputs. Concordance between manual calculations and the software results was examined to ensure the accuracy of the statistical analysis. As there was no difference between manually calculated values and software-generated values, no statistical test was applied.

**Figure 1:**
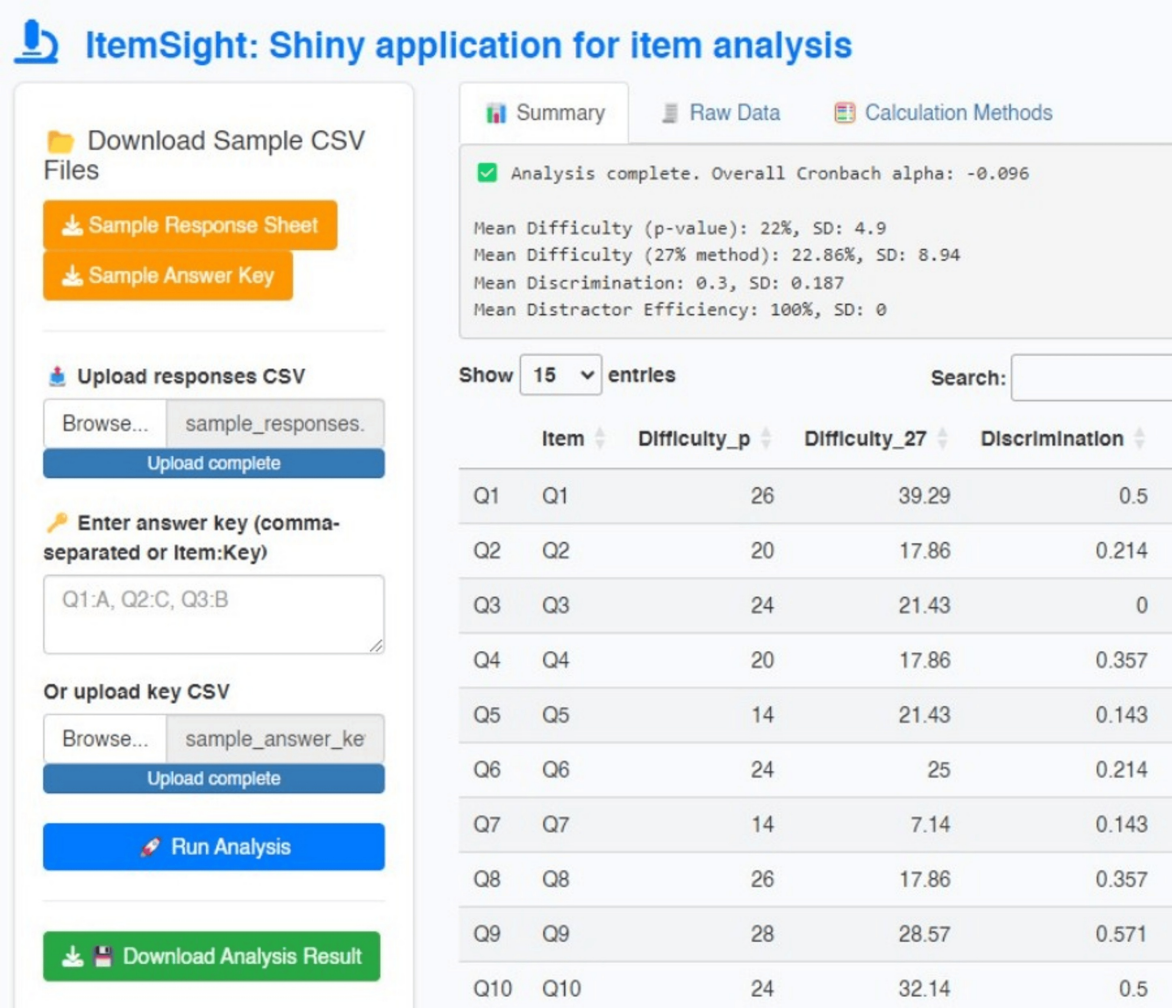
ItemSight: Shiny application for item analysis interface The screenshot displays the user interface of ItemSight, a Shiny-based application designed for automated item analysis of assessment data. The left panel provides options to download sample CSV files (response sheet and answer key), upload student response data, and input the answer key either manually (comma-separated format) or via CSV upload. After data entry, users can execute the analysis using the "Run Analysis" button and download the results. The main panel presents analysis outputs under different tabs, including Summary, Raw Data, and Calculation Methods. The Summary tab (shown) reports key psychometric indices such as overall Cronbach's alpha, mean item difficulty (p-value and 27% method), mean discrimination index, and distractor efficiency. Below the summary, a detailed item-wise table displays parameters. CSV: comma-separated value

The workflow was performed on a standard ASUS Vivobook (X1502ZA) laptop. The system is powered by a 12th Generation Intel Core i3-1215U processor with 16 GB RAM. It includes integrated 128 MB Intel UHD Graphics and 477 GB storage. The device runs on a 64-bit Windows 11 operating system. ItemSight successfully processed (analyzed data without software glitch) all 10 datasets without errors, indicating that the software is capable of handling both moderate and large volumes of data as designed by its underlying algorithm. The analysis was done without any visible time lag. One such output is shown in Figure 1.

Across all tested response sets, the item statistics by the software were fully consistent with those obtained through standard manual calculations. The generated item analysis report can be exported in comma-separated values (CSV) format, allowing users to conduct additional statistical analysis or archival storage as needed. All files, namely, student responses, answer keys, and generated reports, remain stored locally on the user's computer, thereby ensuring data confidentiality and eliminating reliance on external servers.

How to use the application

Before using the ItemSight Shiny application, users need to have R (version 4.0 or above recommended) and RStudio installed on their computer. The app requires several R packages to be installed before running. The application code contains a prompt to install all dependencies. Hence, the first time, it will take some time to install the packages.

**Figure 2:**
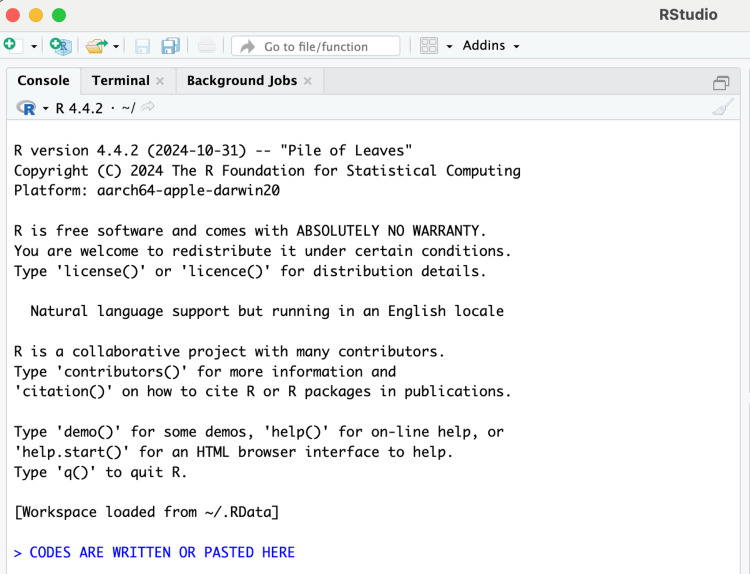
RStudio console showing the place where code is written or pasted from elsewhere The screenshot depicts the console panel of RStudio with R version 4.4.2 ("Pile of Leaves") successfully launched. The interface displays startup information, including platform details, licensing notice, and guidance commands (e.g., help(), citation(), demo()). The console (bottom-left pane) is the primary workspace where users write or paste R code, execute commands, and view outputs. Tabs for Console, Terminal, and Background Jobs are visible, indicating support for interactive coding, system-level commands, and parallel task execution. This environment serves as the foundational workspace for developing scripts, running analyses, and building Shiny applications.

To use ItemSight, users need to open RStudio (Figure 2) and copy the code from the downloaded Microsoft Word document and paste it into the RStudio console and press Enter on a Windows PC or Return on a Macintosh computer.

To use the ItemSight app, the user needs to have two files in CSV format: a spreadsheet containing the students' responses (each row representing a student and each column representing an item) and a corresponding answer key file. The user needs to first download the provided "Sample Response Sheet" and "Sample Answer Key" from within the app (Figure 1), which can be opened in Microsoft Excel or similar spreadsheet applications. The user needs to enter their own student responses in the response sheet and fill in the correct answers in the answer key.

After preparing the files, the user needs to upload the response sheet by clicking the Browse button under "Upload responses CSV", followed by uploading the answer key through the Browse button under "Or upload key CSV". Next, the user needs to click on Run Analysis, after which the results will automatically appear under the Summary tab, showing difficulty indices, discrimination index, point-biserial correlation, distractor efficiency, and Cronbach's alpha (Figure 1). Finally, to save the output, the user needs to click on Download Analysis Result, which will generate a CSV file containing the complete item analysis.

## Discussion

The application offers several advantages for teachers and researchers conducting item analysis. It automates the entire process, allowing users to upload student responses and answer keys and instantly obtain key metrics such as difficulty, discrimination, distractor efficiency, point-biserial correlation, and reliability. This saves time and minimizes calculation errors compared to manual methods. The interface is user-friendly, and teachers only require moderate computer skills and no programming knowledge. The app also allows downloading of results for further review or reporting, making it practical for routine assessment evaluation. These data can also be used for research purposes. In addition, RStudio and this application run on a personal computer, and no data is shared with anyone. Hence, the data security is there.

The current version of the application has some practical limitations. It is designed to handle type A MCQs with fixed options (A-D) [20] and does not support other response formats like three-option or five-option MCQ or partial marking. The app requires properly formatted CSV files for input, and any mismatch in file structure or key entry can lead to errors. It does not include advanced error-checking or automated handling of incomplete or irregular datasets, so the user must ensure data quality before analysis. The app also runs locally in R Shiny, which means performance may slow down when analyzing very large datasets.

We did not include a built-in interpretation facility, as different institutions may adopt varying criteria to categorize MCQs [21,22]. The application provides the raw scores, allowing educators or administrators to make further decisions regarding item quality or classification according to the type of examination and the respective institutional policy.

How to use the application for educational and research purposes

The Shiny application is an open-source application, and anyone can use it for any purpose. No permission is required for using the application for educational purposes. Researchers, while using the application for analysis and publication of the results, can cite this research paper for attribution. For any help, they can contact the corresponding author of the article.

## Conclusions

ItemSight provides a freely accessible platform for conducting item analysis of MCQs. By integrating key metrics such as difficulty index, discrimination index, distractor efficiency, point-biserial correlation, and Cronbach's alpha into a single user-friendly Shiny interface, the application reduces the burden of manual calculation and ensures accuracy in assessment evaluation. Its ability to generate structured outputs and downloadable reports makes it a practical tool for teachers and researchers seeking evidence-based improvement of test items. While it has certain technical limitations, ItemSight offers a valuable first-line solution for routine item analysis and supports the broader goal of enhancing the quality of student assessments through data-driven insights.
